# Child sexual abuse among adolescents in southeast Nigeria: A concealed public health behavioral issue

**DOI:** 10.12669/pjms.314.7115

**Published:** 2015

**Authors:** Manyike Pius C, Chinawa Josephat M, Aniwada Elias, Odutola Odetunde I, Chinawa T. Awoere

**Affiliations:** 1 Manyike Pius C, College of Medicine, Department of Pediatrics, Federal Teaching Hospital Abakiliki, Nigeria; 2 Chinawa Josephat M, College of Medicine, Department of Pediatrics, University of Nigeria/University of Nigeria Teaching Hospital (UNTH), Ituku- Ozalla, Enugu State, Nigeria; 3 Aniwada Elias, Department Of Community Medicine, University of Nigeria Teaching Hospital (UNTH), Ituku- Ozalla, Enugu State, Nigeria; 4 Odutola Odetunde I, College of Medicine, Department of Pediatrics, University of Nigeria/University of Nigeria Teaching Hospital (UNTH), Ituku- Ozalla, Enugu State, Nigeria; 5 Chinawa T. Awoere, Department Of Community Medicine, University of Nigeria Teaching Hospital (UNTH), Ituku- Ozalla, Enugu State, Nigeria

**Keywords:** Child sexual abuse, Adolescents, Nigeria

## Abstract

**Background and Objective:**

Child sexual abuse among adolescents is an often overlooked issue in pediatrics, yet it is a major cause of low self esteem and stigmatization in adolescents. The objective of this study was to determine the socioeconomic determinant and pattern of child sexual abuse among adolescent attending secondary schools in South East Nigeria.

**Methods:**

This was a cross-sectional study that was carried out among children in three secondary schools in Enugu and Ebonyi states of Nigeria. Five hundred and six adolescents who met inclusion criteria were consecutively recruited into our prospective study between June and October, 2014.

**Results:**

One hundred and ninety nine (40 %) of the respondents had been abused and the commonest form of abuse was to look at pornographic pictures, drawings, films, videotapes or magazine 93(18.4%). Fifty eight (11.5%) adolescents stated that they were abused once with age at first exposure being 7-12 years 57 (11.4%). When grouped together, family members and relatives are perpetrators of child sexual abuse. There was significant difference in sex abuse between males and females (p=0.014) while there were no significant difference for age (p=0.157) and social class (p=0.233).

**Conclusion:**

Overall prevalence and one time prevalence rates of sexual abuse among adolescents in south east Nigeria was 40% and 11.5% respectively with male perpetrators. There is no link between socioeconomic class, age and child sexual abuse among adolescents.

## INTRODUCTION

Sexual abuse seen in childhood or adolescence is a debilitating event that can have serious, long-term physiologic and psychosocial effects.^1^ The impact of adolescent sexual abuse is well documented; it contributes immensely in poor school performance, substance abuse, delinquency, prostitution, sexual dysfunction, mental illness, suicide, and transmission of abusive behavior to subsequent generations.^2^-^4^

It is noted that over the past two decades, prevalence estimates of child sexual abuse among adolescents ranges between 6% and 62% for females and 3% and 31% for males.^5^ Incident-Based Reporting System, which provided a data on sexual assault reported to law enforcement agencies, indicates that sexual assault is most prevalent among adolescents in contrast to any other age group, with 33% of all victims falling within the ages of 13–17.^6^ It is important to note, though, that prevalence rates based on reported incidents are likely underestimates of the problem; only half of all adolescent victims will tell anyone about the incident^7^ and only 6% will report the incident to authorities.^8^

Child sexual abuse among adolescent had been found to be influenced by a decline in socioeconomic status, and the disruption of intimate relationships. Adolescent child sexual abuse was more common in those from disturbed and disrupted families and in those who also reported physical and emotional abuse.^9^,^10^ Sexual abuse among adolescents may take many forms and vary in terms of frequency, duration, invasiveness of the acts involved, and the use of force or coercion.^10^

This study was aimed at investigating the prevalence and socioeconomic determinant of child sexual abuse among adolescent attending secondary schools in south east Nigeria. Our hope is that this study will shed light on this topic in both primary and secondary schools in southeast Nigeria and to help parents of affected children cope with the management and prevention of this social problem.

Evaluation of prevalence and pattern of sexual abuse among adolescent is underreported in paediatrics practice and its importance cannot be overemphasized especially its impact on health which include post-traumatic symptoms, precocious sexualization and depression.^11^,^12^

This study is essential for the formulation of policies on awareness, intervention and its impact on adolescents. Much had not been done on this subject in Nigeria in particular and Africa in general. This study will, therefore, help to determine if there is a difference in prevalence. In addition, this study will establish baseline patterns of distribution of adolescent child sexual abuse and will assist physicians to have a high index of suspicion when handling children with these problems.

## METHODS

### Study design

This is a cross-sectional study that assessed the prevalence and socio-demographic pattern of child sexual abuse among adolescents attending secondary schools in Enugu and Ebonyi state, south east Nigeria.

### Study area

The study was carried out among children in three secondary schools in Enugu and Ebonyi states of Nigeria.

### Study population

Five hundred and six adolescents who met inclusion criteria were consecutively recruited into our prospective study between June and October, 2014. Three schools were selected in Enugu and Ebonyi metropolis through simple random sampling method. There were all mixed schools.

### Study Procedure

A structured self-administered questionnaire was used to collect information from the adolescents who attended the selected secondary schools. We used the child sexual abuse questionnaire. The questionnaire has questions on socio economic demographics, type of sexual abuse, experience with sexual abuse, perpetrators of sexual abuse, and frequency of abuse.

Adolescents who signed consent and who understood the questionnaire thoroughly were included in this study, while individuals with psychiatric disorders and adolescents who did not give consent were excluded. Each family was assigned a socioeconomic class using a recommended method, modified by Oyedeji.^13^

### Ethics Statement

This was obtained from the head of the school authorities and government owned institution where this work was carried out.

### Consent

Informed consent was sought from school teachers, parents/caregivers of potential subjects, including older students in secondary schools, before enrolling them into the study.

### Case Selection

Subjects who fulfilled the inclusion criteria were consecutively enrolled into the study.

### Data Analysis

Data was analyzed using the SPSS statistical package, version 17. The chi-square statistical test and T-test were used for categorical and continuous variables, respectively. Data presentation was in tables.

## RESULTS

Majority of respondents 364 (> 70%) were aged 15-19 and of lower socio economic class 236 (about 47%) while they were approximately equal sex distribution. About 199 (40%) of the respondents had been abused and 173 (34.2%) had been spoken to by another youngster on their sex abuse. Table-I

**Table-I:** Socio-demographic variable and Sex abuse exposure.

Variables	Frequency (n=506)	Percent (100)
*Age(Years)*		
10-14	132	26.09
15-19	364	71.94
20-24	10	1.98
*Sex*		
Female	267	52.77
Male	239	47.23
*Social class*		
Upper class	170	33.40
Middle class	100	19.76
Lower class	236	46.64
*Sex abuse exposure*	*Frequency*	*Percent*
Abuse	199	39.3
Never abused	307	60.7
*Knowledge of another youngster who experienced similar events and spoke to you about it*		
Yes	173	34.2
No	333	65.8

The commonest form of abuse was to look at pornographic pictures, drawings, films, videotapes or magazine 93(18.4%), to be naked and to expose their genitals for picture taking or filming 85 (16.8%) and Submission to full sexual intercourse with penetration 74(14.6%). The least form was to watch him/her masturbate 60 (11.9%). Table-II

**Table-II:** Forms of sex abuse.

Variables	Yes	No	No answer
Different ways of sex abuse	n(%)	n(%)	n(%)
Demanding you or forcing you to (look at his/her genitals	81(16.0)	371(73.3)	54(10.7)
Undress and show him/her your genitals	70(13.8)	386(76.3)	50(9.9)
Watch him/her masturbate	60(11.9)	385(76.1)	61(12.1)
Undress with another child and fondle each other in front of him/her	67(13.2)	382(75.5)	57(11.3)
Be fondled (caresses, rubs, kisses on the whole body and/or his/her genitals)	76(15.0)	374(73.9)	56(11.1)
Fondle him/her caresses, rubs, kisses on the whole body and or his/her genitals	82(16.2)	368(72.7)	56(11.1)
Look at pornographic pictures, drawings, films, videotapes or magazine	93(18.4)	360(71.1)	53(10.5)
Be naked and to expose your genitals for picture taking or filming	85(16.8)	371(73.3)	50(9.9)
Submit to full sexual intercourse with penetration	74(14.6)	372(73.5)	60(11.9)
Submit to having his/her fingers or on object introduced in your body	66(13.0)	384(75.9)	56(11.1)

Seventy five(14.8%) of respondents could not remember the number of times it happened to them while 58(11.5%) stated that it happened once. Age at first exposure, were 7-12 years 57 (11.4%) and above 12 years 82(16.2%). Sixty eight (13.4%) were still being abused. Age of respondents at last exposure was 80 (15.8%) for those above 12 years while 31 (6.1%) cannot remember. Most of perpetrators were neighbors 54(27.1%), family friends 39(19.6%) and teachers 30 (15.1%). When grouped together, family members and relatives are perpetrators of child sexual abuse, friends 97(48.7%) and neighbors 74(37.2%). Table-III

**Table-III:** Experience with sexual abuse and perpetuators of sex abuse.

Variables	Frequency	Percent
*Experience with sexual abuse Number of times it happened to you*		
Once	58	11.5
2-5 times	25	4.9
6-10 times	17	3.4
>10 times	24	4.7
can’t remember	75	14.8
Never happened to me	307	60.7
*Your age when it happened the first time*		
≤6 years	29	5.7
7-12 years	57	11.4
>12years	82	16.2
can’t remember	31	6.1
Never happened to me	307	60.7
*Presently still being exposed to sexual abuse*		
Yes	68	13.4
No	131	25.9
Never happened to me	307	60.7
*Those that has stopped, your age the last time it happened to you*		
≤6 years	18	3.6
7-12 years	60	11.9
>12years	80	15.8
can’t remember	41	60.7
Never happened to me	307	60.7
*Variables*	*Frequency*	*Percent*
The person that perpetuated the abuse	n=199	
Stranger	39	19.6
Baby sitter	20	10.1
Family friend	39	19.6
Neighbor	54	27.1
Teacher	30	15.1
Instructor	15	7.5
Peer	35	17.6
Father or Mother	33	16.6
Step father or Stepmother	18	9.0
Mother’s or Father’s friend	23	11.6
Brother or Sister	23	11.6
Half-brother or Half sister	19	9.5
Uncle or Aunt	19	9.5
Grandfather or Grand mother	17	8.5
Someone else	32	16.1
Perpetuators of sex abuse (Groups)	n=199	
Family members	127	63.8
Friends	97	48.7
Neighbors	74	37.2
Teacher/Instructor	45	22.6
Others	71	35.7

Association between socio-demographic variables, sex abuse experience and regression on socio-demographic variable and sex abuse experience. There was significant difference in sex abuse between males and females (p=0.014) while there were no significant difference for age (p=0.157) and social class (p=0.233). It also shows that those aged 15-19 were 1.33 times and those aged 20-24 years 1.04 times less likely to be abused. Females were 1.53 times more likely to be abused than males. Those from middle class and lower class were 1.09 and 1.14 times more likely to be abused than higher class. Table-IV

**Table-IV:** Association between socio-demographic variable and sex abuse experience and regression on socio-demographic variable and sex abuse experience.

Socio-demographic variable	OR	Sig.	95% C.I. for OR	
			Lower	Upper
*Age*				
10-14				
15-19	0.755	0.680	0.199	2.868
20-24	0.962	0.954	0.263	3.521
*Sex*				
Female	1.525	0.028	1.046	2.224
Male				
*Social class*				
Upper class				
Middle class	1.092	0.684	0.714	1.671
Lower class	1.140	0.601	0.697	1.866

Socio-demographic variable	Abused	Not abused	Test Statistics Χ^2^	p value
*Age*				
10-14	43(32.6)	89(67.4)		
15-19	151(41.5)	213(58.5)	3.708	0.157
20-24	5(50.0)	5(50.0)		
*Sex*				
Female	91(34.1)	176(65.9)	6.519	0.014
Male	108(45.2)	131(54.8)		
*Social class*				
Upper class	58(34.1)	112(65.9)		
Middle class	42(42.0)	58(58.0)	2.913	0.233
Lower class	99(41.9)	137(58.1)		

## DISCUSSION

We noted the overall prevalence and one time prevalence rates of child sexual abuse among adolescents as 40% and 11.5% respectively. These prevalence rates are similar to that of Meichun et al.^13^ who reported prevalence of 35.1 and 14.9% respectively. The similarity of high prevalence rates in this study and that of Meichum despite different methodology used in both studies highlights the burden and emerging trends of adolescents’ sexual abuse. It is pertinent to note that Chinawa et al.^14^ reported a prevalence rate of 10.5%. We believe that the prevalence could be higher if not for poor reporting.^15^,^16^ However, it is important to note that variations in adolescent sexual abuse across geographical areas either could reflect true differences in incidence or could be affected by how disclosure and reporting of cases are experienced in different cultures. Notwithstanding, the reasons for non-reporting are complex and multifaceted.^17^ These reasons may include a number of factors such as the age of the abused child, the relationship between the perpetrator and the abused, the likely consequences of the disclosure.^18^

We also noted with interest several forms of child sexual abuse among adolescents. Forcing adolescents to watch pornographic pictures, drawings, films, videotapes or magazine, perpetrators exposing their nakedness and genitals for picture taking or filming, full sexual intercourse with penetration and perpetrators forcing their victims to watch them masturbate are several forms of abuse noted in this study.

The most commonly reported form was sexual abuse without physical contact, whereas sexual abuse with penetration was least commonly reported. Meichum et al.^13^ and Tracy^19^ independently reported similar findings. The reason for this high frequency of non physical contact of sexual abuse could be because adolescent sexual abuse without physical contact happened most frequently at home or in cyberspace, and perpetrators were most often strangers. Child sexual abuse among adolescents with physical contact happened most frequently in a public place or a house other than the victim’s, and most perpetrators were known to the victims.

It is important to point out here the impact of pornography as a form of adolescent sexual abuse in this study. There are lots of studies about the effects of pornography on adolescents. There have been studies that seem to demonstrate arousal of perpetrators from viewing child pornography, particularly pedophile. It is not known if pornography makes them act on their arousal, but it seems to be part of the constellation about what causes them to abuse.^20^

It’s also found that viewing violent or sexual materials can affect attitudes involving adult rape. There is also a link between viewing violent pornography and repeat abuse by perpetrators, however the causation between the two is not known.^20^

A tenth of adolescents in this study were sexually abused once while twice that number had repeated abuse up to ten times. This finding conforms to a study in USA, where every 2 minutes, an adolescent is sexually assaulted; more over an average of 237,868 victims of sexual assault occur each year.^21^ It has been documented that the average duration of physical and sexual abuse was 5 and 2 years, respectively.^22^ The abuse can go on and on for many years, well into adolescence or even adulthood. There are reported cases of fathers sexually abusing daughters into adulthood.^22^

We noted that the age at first sexual exposure occurred more for those above 12 years and least between 7-12 years.^22^ This is also similar to a study where age of first abuse was before the age of 17 years, with age of unwanted experience ranging between 6 and 18 years. However a contrary report showed that the average age for first abuse is 9.9 years for boys and 9.6 years for girls.^23^ The reason for this conflicting reportage could be due to different cultural and religious background.

Surprisingly, majority of victims reported family members and relatives as perpetrators. It is noted that adolescent sexual abuse can take place within the family, by a parent, step-parent, sibling or other relative. Majority of the perpetrators are males. Studies using the law enforcement as well as victim self-report data found that more than 90% of the perpetrators of sexual offenses against minors were males.^24^

This study also revealed that females were 1.53 times more likely to be abused than males. Females were four times more likely to report sexual abuse than males. It is also noted that 1 in 4 girls (25%) are sexually abused by the age of 18. One in 6 boys (17%) are sexually abused by the age of 18. Other studies have also documented female preponderance.^25^ The vulnerability of a female child and how the society treats a female child with disdain and levity all explain this female preponderance.

This study also showed that males are also abused from this study. Though we did not study the impact of sexual abuse on the adolescent, it is important to note that male adolescent victims of sexual abuse experienced more difficulties in school, marijuana use, delinquent behavior, and sexual risk taking behavior as compared to female victims.^26^

We noted no correlation between social class, age and sexual abuse in adolescents. This is in tandem with the work of Ismail et al. in a cross sectional study of six countries who noted no associations with demographic characteristics.^27^ This is also supported by Sentelli and his colleagues in a different study with different methodology.^28^

## CONCLUSION

We conclude that overall prevalence and one time prevalence rates of sexual abuse among adolescents in south east Nigeria as 40% and 11.5% respectively with male perpetrators. There is no link between socioeconomic class, age and child sexual abuse among adolescents.
